# Comparison of Syringes With Intravitreal Anti-VEGF Drugs: Particle Burden and Protein Aggregates in Brolucizumab, Aflibercept and Bevacizumab

**DOI:** 10.1167/tvst.10.9.21

**Published:** 2021-08-18

**Authors:** Marc Schargus, Katharina Tatjana Kopp, Constanze Helbig, Andreas Frings, Gerhard Winter

**Affiliations:** 1 Department of Ophthalmology, Heinrich Heine University Düsseldorf, Düsseldorf, Germany; 2 Department of Ophthalmology, Asklepios Hospital Nord-Heidberg, Hamburg, Germany; 3 Department of Pharmacy, Pharmaceutical Technology and Biopharmaceutics, Ludwig-Maximilians-University Munich, Germany; 4 Coriolis Pharma Research GmbH, Martinsried, Germany

**Keywords:** aflibercept, bevacizumab, brolucizumab, contamination, protein particles, silicone oil, intravitreal injection

## Abstract

**Purpose:**

In a benchwork particle counting analytical evaluation, the number and type of particles in intravitreal injection formulations of three different agents against vascular endothelial growth factor were investigated.

**Methods:**

Commercially available ready-to-use aflibercept and brolucizumab glass syringes, vials containing bevacizumab (off-label use in ophthalmology), and repackaged ready-to-use plastic syringes containing bevacizumab were tested without filtration. Total visible, subvisible, and nanoparticles numbers and size distributions were quantified using light obscuration, flow imaging, resonant mass measurement (RMM), tunable resistive pulse sensing, and dynamic light scattering.

**Results:**

Repackaged bevacizumab showed overall low particle numbers, aflibercept showed high numbers of micrometer sized particles but low nanoparticle numbers, brolucizumab showed low to moderate numbers of micrometer sized particles but high nanoparticle numbers. RMM measurements identified particles in the nanometer range as either proteinaceous or silicon oil; the nature of the other particles was not further evaluated.

**Conclusions:**

Repackaged bevacizumab shows no inferior particle quality compared to ready-to-use products. It is relevant to study nanoparticle load of the products as the micrometer-sized particle numbers do not in all cases correlate to nanoparticle counts. Particularly for the high concentration product Beovu (brolucizumab), high nanoparticle numbers were found despite low numbers of micrometer sized particles. Silicone oil droplets did not account for high particle numbers as the measured numbers were low.

**Translational Relevance:**

Different side effects are registered in different frequencies with different intravitreal anti-VEGF-drugs and syringes, which are applied by injection by small 30G needles through the sclera directly to the intravitreal cavity. The study of nanoparticles and silicone oil droplets may be able to contribute to narrowing down the causes.

## Introduction

Since the first reports of the use of anti-VEGF agents for the treatment of retinal vascular diseases in 2005, a variety of drugs have been developed.^1^ These include ranibizumab (Lucentis; Genentech, San Francisco, CA), aflibercept (Eylea; Regeneron Pharmaceuticals, Inc., Tarrytown, NY), bevacizumab (Avastin; Roche Pharma AG, Grenzach-Whylen, Germany), and brolucizumab (Beovu; Novartis Europharm Limited, Dublin, Ireland). All these agents have demonstrated high efficacy to reduce vision loss and improve visual acuity in different retinal vascular diseases, mainly choroidal neovascularization due to age-related macular degeneration. Bevacizumab (Avastin) is a recombinant monoclonal IgG1 antibody with a molecular weight of 149 kDa.^2^ Avastin can be purchased in glass vials with a protein concentration of 25 mg/mL.^2^ For intravitreal injection, the drug is drawn into a syringe by compounding pharmacies and stored at 2°C to 8°C until further usage. Volumes differ from 50 to 100 µL or even up to 150 µL.^3^^–^^5^ The use of bevacizumab for intravitreal injections is a well- known off-label use in ophthalmology.

Aflibercept (Eylea) is a dimeric glycosylated recombinant fusion glycoprotein with a molecular weight of 115 kDa.^6^^–^^8^ Dosage forms of Eylea are either a single-dose glass vial of 0.1 mL content or a single-dose-prefilled syringe from the manufacturer with 90 µL, both used to deliver 50 µL of drug solution (40 mg/mL).^9^

Brolucizumab is the active ingredient in Beovu. It is a recombinant single-chain Fv antibody fragment of 26 kDa and exhibits high affinity to VEGF A.^10^ It is available both as a glass vial and a single-use prefilled syringe with 0.165 mL, delivering 50 µL of protein solution with a concentration of 120 mg/mL.^11^ The concentration of the active ingredient is 3 to 12 times higher than that of the other agents.

Long-term or sustained intraocular pressure (IOP) rise has been reported clinically in several case reports and case series after multiple intravitreal injections. Retrospective analysis of the VIEW 1 and 2 data of 2457 patients showed statistically significant higher IOP elevation in ranibizumab than in aflibercept injected eyes.^12^ Notably all studies evaluating IOP elevations after anti-VEGF injections are limited because of the retrospective design of the analysis. Several mechanisms have been proposed to explain clinically significant IOP elevation after anti-VEGF treatment, including protein aggregates and silicone oil droplets as reasons for blocking trabecular meshwork outflow.^13^^,^^14^ The United States Pharmacopeia (USP) sets clear limits concerning particulate matter in ophthalmic solutions in <789>. Particle numbers are not allowed to exceed 50 particles/mL, ≥10 µm, 5 particles/mL, ≥25 µm, and 2 particles/mL, ≥50 µm.^15^ If particle numbers are higher, the product is not suitable for patient application.

Now there are a number of published cases of severe visual acuity loss associated with intravitreal inflammation, retinal vasculitis, and retinal artery occlusion after treatment with brolucizumab.^16^^–^^22^ The reported incidences in the HAWK and HARRIER studies for definite uveitis was 4.6% for brolucizumab and only 1.1% for aflibercept.^18^ Williams et al. reported low rates of noninfectious vitritis of only 0.1% after 66,356 injections of bevacizumab, 0.02% after 26,161 ranibizumab injections, and 0.16% after 8071 aflibercept injections.^23^ There are several theories regarding why this rare but potentially serious complication may occur. The cases of noninfectious uveitis that occurred after anti-VEGF injection in the period before brolucizumab entered the market partly took place in clusters.^24^ It was postulated that this phenomenon occurred in part only with certain syringes that were shown to have increased silicone oil droplet content or after flicking of the syringes.^25^ The cause of the increased rate of noninfectious uveitis and retinal complications has not yet been determined.

Beside type IV hypersensitivity reactions after intravitreal brolucizumab along the vessel walls, there are several other conceivable theories.^16^

In a previous study we evaluated in vitro the particle levels in anti-VEGF syringes with bevacizumab, ranibizumab, and aflibercept from different compounding pharmacies and the first ready-to-use syringe for ranibizumab.^4^ All three tested agents in this earlier study were available in similar quality regarding particulate purity and silicone oil microdroplet count. Repackaging in this study had a major impact on the quality. In the meantime, a prefilled syringe of aflibercept is also available and has widely displaced the use of the vial. The recently approved agent brolucizumab is also predominantly available as a prefilled syringe.

The aim of this study was to add further laboratory data on levels of subvisible particles and protein aggregates in the new prefilled aflibercept and brolucizumab syringes and to compare it to samples of bevacizumab delivered from a compounding pharmacy.

## Materials and Methods

### Materials

All products implemented in the study were sent to the place of measurement under cooling conditions and stored at 4°C after arrival. Avastin (10 syringes, 3.75 mg bevacizumab/150 µL) was repackaged by Asklepios Klinik Nord, Hamburg into 1 mL BD Plastipak Luer-Lock syringes (Becton, Dickinson and Company, Franklin Lakes, NJ) and in total kept for a maximum of five days. Avastin was provided in glass vials (4 mL, 25 mg/mL). Eylea (40 mg aflibercept/mL) and Beovu (120 mg brolucizumab/mL) were both obtained as prefilled syringes from the manufacturer with 90 µL and 165 µL each, respectively.

Depending on the small number and volume of the samples, different pools had to be formed, as indicated in Table 1.

**Table 1.** Sample Names, Concentration, Primary Packaging, and Number of Pools and Aliquots Formed at LMU Munich

Sample Name	Concentration mg/mL	Volume Per Container	Primary Packaging	Repackaged	Pooled Samples
Avastin	25.00	4 ml	Vial	No	4 aliquots with 800 µL were taken from one vial
Avastin	25.00	150 µl	Syringes	Yes	2 pools with 750 µl were formed from 5 syringes each
Eylea	40.00	90 µl	Syringes	No	1 pool with 720 µl was formed from 8 syringes
Beovu	120.00	165 µl	Syringes	No	2 pools with 660 µl were formed from 4 syringes each

None of the products of the study were filtrated before pools were formed or aliquots were aspired. To form two pools from repackaged Avastin (Avastin 1 and 2), five syringes each were discharged into two 15 mL Greiner polypropylene (PP) tubes.

One pool was formed by discharging eight syringes of Eylea into one 15 ml Greiner PP tube.

Two pools of Beovu (Beovu 1 and 2) were prepared discharging four syringes each into two 15 mL Greiner PP tubes (Greiner tubes from Sigma-Aldrich Inc., Darmstadt, Germany).

From the comparably large Avastin vial, aliquots of 0.8 ml each were aspired with a sterile 18G × 1 ½ needle into four sterile 1 mL Luer-Lock Tip syringes (both Becton, Dickinson and Company). Each syringe was discharged into a 15 mL Greiner PP tube to form four samples (Avastin vials 1–4).

Due to the small volume of the available products, the products had then to be diluted to allow all analytical measurements. All products were diluted 1:20 with the corresponding product buffer (placebo) solutions. The corresponding buffer solutions of the marketed products were used to avoid any incompatibility with other diluents was taken from official documents.^2^^,^^9^^,^^10^ There were no gas bubbles inside the evaluated syringes.

### Measurement Techniques

All measurements were performed on calibrated equipment: FlowCAM and PAMAS were calibrated with Duke Standards and Count-Cal Particle Count Controls (NIST Traceable Size Standards 2, 10, and 25 µm) (all Thermo Fisher Scientific, Fremont, Waltham, MA). Different measurement techniques were used to assess particle sizes and numbers. To detect subvisible and visible particles, light obscuration was used, in principle following USP guidelines. As orthogonal technique, flow imaging was applied, which is able to detect translucent particles in the subvisible size range better than light obscuration. To measure particles in the nanometer range, resonant mass measurements and tunable resistive pulse sensing were applied. RMM also allowed the differentiation of protein and silicon oil particles by their difference in densities.

### Nephelometry

Turbidity was determined with the NEPHLA turbidimeter (Dr. Lange, Düsseldorf, Germany). A sample volume of 1.5 mL was measured in triplicates at 860 nm and results were given in formazin nephelometric units (FNU).

### Flow Imaging

Particles in the micrometer range, were analyzed using the FlowCAM 8100 (Fluid Imaging Technologies, Inc., Scarborough, ME) with a 10× magnification cell (80 µm × 700 µm). The flow cell was rinsed, and cleanliness was verified with highly purified water (HPW, <100 particles/mL). Samples were measured in triplicates of 150 µL at a flow rate of 150 µL/min and a threshold of 1013. Results were evaluated with the software Visual Spreadsheet Version 4.7.6 (Fluid Imaging Technologies, Inc.).

### Light Obscuration (LO)

The PAMAS SVSS-C system (PAMAS GmbH, Rutesheim, Germany) was used to quantify particles in the subvisible range following USP methodology. Cleanliness was verified (<20 particles/mL HPW) and each measurement consisted of a prerun of a 400 µL sample, followed by a triplicates of 300 µL. Obtained results were analyzed using the implemented software PAMAS PMA program V 2.1.2.0 (PAMAS GmbH).

### Resonant Mass Measurements (RMM)

An Archimedes system, equipped with a Hi-Q Micro Sensor (both Malvern Instruments, Malvern, UK) and the Archimedes software v1.20 was used for RMM analysis of particles up to 4 µm in size. The system was calibrated with polystyrene size standards of 0.994 µm specified diameter (Duke Standards; Thermo Fisher Scientific, Waltham, MA) and system cleanliness was verified.

Particle densities were set to 0.97 g/mL for positively buoyant particles (considered as silicone oil particles), and to 1.32 g/mL for negatively buoyant particles (considered as proteinaceous). Each sample was loaded for 40 seconds and the limit of detection was automatically determined by the instrument software. Minimum detectable particle sizes were approx. 485 nm for silicone oil particles and approx. 274 nm for protein particles.

Samples were analyzed in triplicates with a measurement time of 600 seconds as stop criterion, corresponding to an analyzed volume of ∼150 nL per replicate.

Data evaluation was performed with the LINK software platform v2.3.22.200619 (Lumetics, Nepean, ON, Canada).

### Tunable Resistive Pulse Sensing (TRPS)

Particles in the nanometer range (150–900 nm) were determined with TRPS using the qNano Gold (IZON, Nottingham, UK). For analysis, pore NP 300 (A57745, IZON) was chosen, a stretch of 47.01 mm and a pressure of around 15 mbar were applied. All samples, buffers, and the calibration particles CPC 400 (mean diameter 350 nm, 7.56 × 10^8^ mg/mL; IZON) were spiked with a small amount of NaCl solution up to a final concentration of 140 mM of sodium chloride.

The lower fluid chamber was loaded with 80 µL of the respective buffer and the voltage was set to 0.34 V for the analysis of the Avastin vial and to 0.38 V for all remaining samples.

Twenty-five microliters of spiked sample or buffer were pipetted into the upper cell and measured in triplicates for 10 minutes. Results were calculated using the included software IZON CONTROL SUITE (IZON), with the focus on particle sizes of 150 and 300 nm.

To ensure no aggregation occurred due to spiking the samples, size and polydispersity (PDI) were counterchecked with dynamic light scattering for all samples used for TRPS measurements.

### Dynamic Light Scattering (DLS)

To determine size and PDI, 25 µL of each sample was pipetted in triplicates in a 384 well plate (Corning, Glandale, AZ). The plate was spun down at 1000 rpm for one minute at 20°C with the Heraeus Megafuge 40 centrifuge with a M20-well plate rotor (both Thermo Fisher Scientific), each well was sealed with 5 µL of silicone oil and centrifuged again. Then it was measured at 25°C with 10 acquisitions of five seconds for each well using the DynaPro DLS plate reader (Wyatt Technology Europe, Dernbach, Germany) and results were calculated using the included Dynamics V7.8 software.

## Results

Results for DLS (size, PDI) and turbidity measurements are presented for the diluted protein solutions (1:20 dilution) as measured. All other results are reported as calculated particle numbers per milliliter for the commercially available, undiluted drug solution.

### Nephelometry

All samples showed low turbidity values between 0.86 and 1.03 FNU. No distinct differences or trends between the products were found.

### Flow Imaging (FlowCAM) and Light Obscuration (LO) Particle Counting

Table 2 provides an overview of all particle numbers measured with FlowCAM and LO.

**Table 2.** Calculated, Cumulative Particle Numbers Per Milliliter (≥1, ≥10, and ≥25 µm) of Samples and Buffers, Measured with LO and FlowCAM

	Light Obscuration [Particles/mL] (Calculated)			FlowCAM [Particles/mL] (Calculated)		
	≥1 µm	≥10 µm	≥25 µm	≥1 µm	≥10 µm	≥25 µm
Buffer A. vial	6 ± 2	0 ± 0	0 ± 0	21 ± 0	0 ± 0	0 ± 0
Avastin vial 1	27,948 ± 9817	207 ± 34	0 ± 0	15,047 ± 2167	200 ± 0	0 ± 0
Avastin vial 2	20,918 ± 7162	163 ± 110	0 ± 0	44,640 ± 2160	2960 ± 428	207 ± 358
Avastin vial 3	12,904 ± 1671	185 ± 68	0 ± 0	39,780 ± 1828	893 ± 316	67 ± 115
Avastin vial 4	42,911 ± 521	126 ± 56	0 ± 0	319,727 ± 6916	2133 ± 780	133 ± 115
Buffer A.	9 ± 5	1 ± 1	0 ± 0	14 ± 16	0 ± 0	0 ± 0
Avastin 1	6281 ± 965	237 ± 161	0 ± 0	30,660 ± 5148	0 ± 0	0 ± 0
Avastin 2	7007 ± 219	244 ± 309	0 ± 0	33,147 ± 22,046	0 ± 0	0 ± 0
Buffer Eylea	23 ± 9	1 ± 1	0 ± 0	623 ± 42	0 ± 0	0 ± 0
Eylea	56,237 ± 7871	74 ± 68	0 ± 0	474,540 ± 27,687	10,600 ± 2462	893 ± 316
Buffer Beovu	137 ± 61	1 ± 1	0 ± 0	100 ± 48	0 ± 0	0 ± 0
Beovu 1	10,615 ± 3658	111 ± 80	0 ± 0	67,840 ± 6803	967 ± 316	133 ± 115
Beovu 2	10,541 ± 2463	193 ± 134	0 ± 0	23,047 ± 1894	413 ± 410	0 ± 0

For both methods all the dilution buffers showed very low particle numbers and must not be considered further.

Looking at the four aliquots from the commercially available Avastin product vial, particle numbers and sizes varied noticeably for both LO and FlowCAM. Avastin vial aliquot 1 exhibited quite low particle counts, whereas vial aliquot 4 showed high numbers for small (≥1 µm) and medium (≥10 µm) particle sizes.

Repackaged bevacizumab syringes showed overall smaller particles numbers than the samples from the Avastin vial. The two pools showed almost identical particle numbers indicating a robust particle level.

Eylea syringes showed very high particle numbers in the small subvisible size range ≥ 1 µm in both applied methods. Particle numbers in the ≥10 and ≥25 µm range were measured low with LO and rather high with FlowCAM.

Beovu syringes showed medium particle levels, comparable to those measured for bevacizumab, falling between the repackaged syringes and the samples from the Avastin vials.

### Resonant Mass Measurements (RMM)

**Figure 1.**
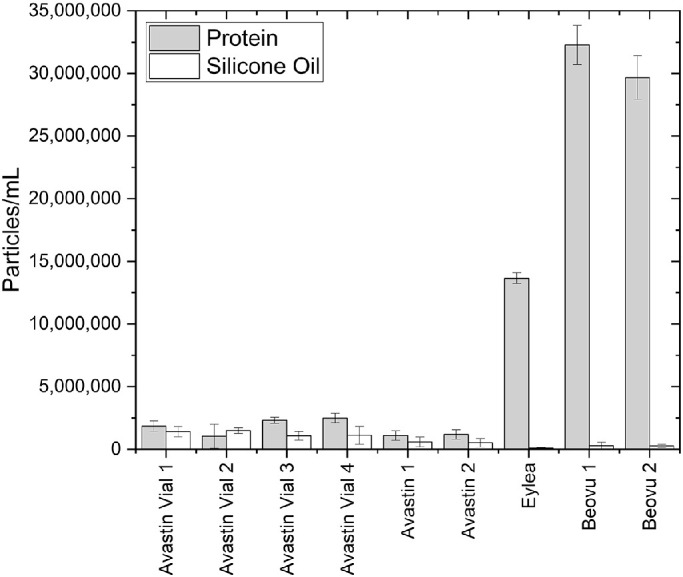
Calculated results from RMM of all samples.

In all RMM measurements only low particle counts near the limit of quantification (3 × 10^5^ particles/mL) were found. With that, calculated numbers for the undiluted products can only be considered as estimates. Particle numbers in the dilution buffers can be considered as negligible. The results for RMM nanoparticle quantification are presented in Figure 1 and Table 3.

**Table 3.** Calculated Results from RMM of All samples and Respective Buffers

	Protein [Particles/mL]	Silicone Oil [Particles/mL]
Buffer A. vial	24,296 ± 7205	0 ± 0
Avastin vial 1	1,850,936 ± 407,138	1,405,230 ± 419,003
Avastin vial 2	1,027,200 ± 959,543	1,489,192 ± 222,570
Avastin vial 3	2,306,753 ± 249,866	1,064,768 ± 343,799
Avastin vial 4	2,497,476 ± 368,782	1,115,095 ± 719,796
Buffer A.	60,237 ± 34,515	24,072 ± 17,049
Avastin 1	1,093,239 ± 378,966	587,901 ± 395,258
Avastin 2	1,184,182 ± 383,312	512,951 ± 345,605
Buffer Eylea	36,770 ± 18,469	3248 ± 5625
Eylea	13,652,825 ± 436,454	87,237 ± 75,553
Buffer Beovu	42,491 ± 25,769	0 ± 0
Beovu 1	32,269,160 ± 1,564,082	278,666 ± 281,964
Beovu 2	29,667,415 ± 1,731,926	266,032 ± 122,289

For all the bevacizumab preparations, only very low nanoparticle numbers were recorded. The number of particles considered as silicone oil droplets was apparently in the same, very low order of magnitude as the protein particle numbers.

Interestingly, for Eylea and for the Beovu pools, comparably higher numbers of about 14 million counts/mL and approx. 29–32 million counts/mL were calculated in the size range between approx. 274 nm and approx. 1000 nm. In none of these cases did silicone oil droplets show up in relevant numbers.

### Tunable Resistive Pulse Sensing (TRPS)

Like for RMM, the overall particle counts were also very low; the particle rate during the measurements was below 100 particles/min.

**Figure 2.**
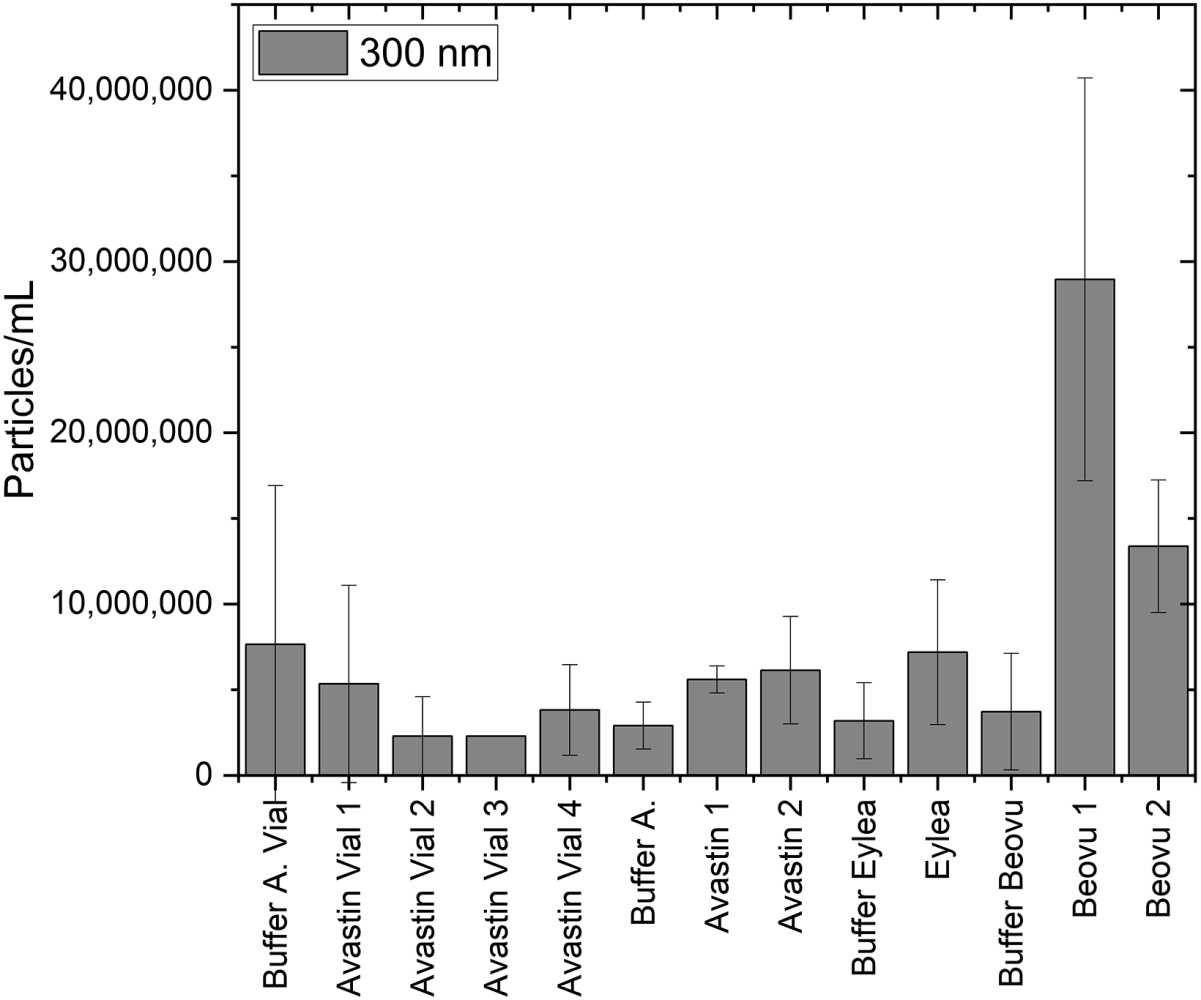
Calculated particle counts (bin size 300 nm) of all buffers and samples measured by TRPS.

The results for the calculated particle numbers at bin sizes of 300 nm are presented in Figure 2 (results for 150 nm are similar, data not shown).

For the bevacizumab products, the nanoparticle numbers for the dilution buffers fell into the same range as for the diluted product samples, indicating that there is no relevant nanoparticle load present.

For Beovu, the situation resembles the same impression received from RMM, that is, having a higher nanoparticle number than for all the other products. The differences between the noise level represented by the buffers and the measured values for the Beovu product was not as large as for RMM. For Eylea, the values found with TRPS are very low, not different from the buffer.

### Size and PDI

Before TRPS measurements, size (hydrodynamic diameter) and PDI of the protein colloids was measured to preclude aggregation due to spiking the samples with 140 mM NaCl. Comparing sizes of normal and spiked samples, no significant differences were seen.

## Discussion

It is well known that anti-VEGF drugs injected intravitreally can long-term increase in IOP.^26^^–^^31^ One commonly discussed potential factor resulting in elevated IOP might be the presence of protein aggregates and silicone oil microdroplets in such drug products.^13^^,^^14^^,^^32^^–^^34^ Experiments in the field of glaucoma research have already clearly demonstrated that the secondary intraocular pressure increase can be due to mechanical obstruction of the trabecular meshwork; this mechanism is also clinically known in ghost cell glaucoma.^35^ Our experimental work aims to complement the previously published study of our group (2018) with data on the newly established ready-to-use syringes and new agents in the field of intravitreal anti-VEGF therapy.^4^

### Comparison of Aliquots from a Commercially Available Avastin Vial and Pools from Repacked Syringes from a Compounding Pharmacy

In the course of repackaging bevacizumab from vials into syringes for intravitreal application handling errors and stresses can be applied to the protein and the used syringes could potentially contain silicone oil that could be shed into the product during handling and intermediate storage.^3^^,^^25^^,^^36^^,^^37^ In this study we cannot confirm previously found quality gaps for repackaged bevacizumab.

The results obtained from LO and FlowCAM show that the repackaged syringes exhibited a low particle count even compared to the samples from the vial regarding particles ≥ 1 µm.

The partly higher particulate burden in aliquots from the vial might be explained by the fact that this material was analyzed as provided, whereas in the compounding pharmacy, particles should have been removed by a filtration step in the course of the repackaging. All particle counts (≥10 µm) obtained by LO measurements would exceed limits set by the USP—counts for particles ≥ 25 and ≥ 50 µm were in line.

### Comparison of Avastin, Beovu, and Eylea

Avastin, both in vial and syringes, contains the lowest concentration of protein (25 mg/mL), Eylea a medium concentration (40 mg/mL) and Beovu the highest (120 mg/mL). Assuming an injection amount of 50 µL per eye, the overall dose of protein (in mg) applied thereby differs by a factor of about 3 to 5 between the products. When we try to correlate the particle numbers found with the protein concentration, we find that Eylea surprises with unexpectedly high numbers of particles in the range of 1 µm. For the larger particles of ≥10and ≥25 µm in Eylea, the dataset is not as clear, because the LO and the FlowCAM numbers differ. The low number for Eylea in LO and the high numbers for FlowCAM indicate that the aggregates formed are of a translucent and (for LO) hard to detect nature. Although being slightly higher in particles than bevacizumab, Beovu does not show a correlation of protein concentration and particle counts. Taking USP <789> into account, all three products would exceed the limit of particle counts set for particles ≥ 10 µm.

Nanoparticles provide a totally different picture. Here, Beovu shows relatively high numbers of nanoparticles, whereas the other products are low to very low. One must consider that the absolute values for Beovu are still low, but it is apparent that here the nanoparticle load correlated with the protein concentration. Eylea (with a medium high protein concentration of 40 mg/ml) is positioned in the middle, at least for one of the two methods applied.

Another observation is also remarkable, that is, that silicone oil nanoparticles play no relevant role in any of the products on the nanoparticulate level.

As mentioned in the introduction, Beovu is the ophthalmic product in the panel studied that contains the smallest therapeutic protein (brolucizumab exhibits a size of only 26 kDa), but with the highest concentration^12^: It is therefore not possible that the nanoparticle counts were caused by high concentrations of the monomeric drug; it must be aggregates of any kind.

### Influence of Particles on the Trabecular Meshwork

So far, it is not known whether nano- or microparticles or the protein colloids themselves could cause a blockage of the trabecular meshwork.

### Rise of Ocular Inflammation

Several reports on the occurrence of mild as well as severe intraocular inflammation up to retinal vaso-occlusion with severe visual loss correlated to the application of the latest anti-VEGF compound brolucizumab have been published in the last year.^18^^,^^19^ A clear explanation for this phenomenon does not yet exist; various theories have been built around the field of hypersensitivity reactions.^38^ Regarding particle size and particle number, it can be noted that the amount of nanoparticles is significantly higher for the brolucizumab product compared to the other agents, whereas for larger particles no relevant difference or even lower number (versus Eylea) are noted. Whether the high concentration of the active agent brolucizumab contributes to the increased number of retinal vaso-occlusive inflammations cannot be drawn from the analyzed data; further analyses in the field of immunoreactions are necessary.

## Conclusions

Repackaged bevacizumab syringes show no quality compromise regarding particles when compared to Avastin from commercially available vials or the ready-to-use syringe products. For subvisible particles rather high numbers for ≥ 1 and ≥ 10 µm were found for all products studied, but these numbers were not correlated to their overall protein concentrations. In contrast, nanoparticle levels were highest in Beovu and correlate to its high protein concentration. The impact of nanoparticles in ophthalmic solutions should be surveilled to make sure they are not the cause of IOP rise or other eye-related diseases, such as intraocular inflammation. Additional limits concerning smaller particles might also be considered for the Pharmacopeia to ensure even higher product quality and safety.
